# Changes in the Brain Endocannabinoid System in Rat Models of Depression

**DOI:** 10.1007/s12640-017-9708-y

**Published:** 2017-02-28

**Authors:** Irena Smaga, Joanna Jastrzębska, Magdalena Zaniewska, Beata Bystrowska, Dawid Gawliński, Agata Faron-Górecka, Żaneta Broniowska, Joanna Miszkiel, Małgorzata Filip

**Affiliations:** 1 Department of Toxicology, Faculty of Pharmacy, College of Medicum, Jagiellonian University, Medyczna 9, PL 30-688 Kraków, Poland; 2 Laboratory of Drug Addiction Pharmacology, Institute of Pharmacology, Polish Academy of Sciences, Smętna 12, PL 31-343 Kraków, Poland; 3 Laboratory of Biochemical Pharmacology, Institute of Pharmacology, Polish Academy of Sciences, Smętna 12, PL 31-343 Kraków, Poland

**Keywords:** Animal model of depression, Endocannabinoid system, Olfactory bulbectomy, Wistar Kyoto

## Abstract

A growing body of evidence implicates the endocannabinoid (eCB) system in the pathophysiology of depression. The aim of this study was to investigate the influence of changes in the eCB system, such as levels of neuromodulators, eCB synthesizing and degrading enzymes, and cannabinoid (CB) receptors, in different brain structures in animal models of depression using behavioral and biochemical analyses. Both models used, i.e., bulbectomized (OBX) and Wistar Kyoto (WKY) rats, were characterized at the behavioral level by increased immobility time. In the OBX rats, anandamide (AEA) levels were decreased in the prefrontal cortex, hippocampus, and striatum and increased in the nucleus accumbens, while 2-arachidonoylglycerol (2-AG) levels were increased in the prefrontal cortex and decreased in the nucleus accumbens with parallel changes in the expression of eCB metabolizing enzymes in several structures. It was also observed that CB_1_ receptor expression decreased in the hippocampus, dorsal striatum, and nucleus accumbens, and CB_2_ receptor expression decreased in the prefrontal cortex and hippocampus. In WKY rats, the levels of eCBs were reduced in the prefrontal cortex (2-AG) and dorsal striatum (AEA) and increased in the prefrontal cortex (AEA) with different changes in the expression of eCB metabolizing enzymes, while the CB_1_ receptor density was increased in several brain regions. These findings suggest that dysregulation in the eCB system is implicated in the pathogenesis of depression, although neurochemical changes were linked to the particular brain structure and the factor inducing depression (surgical removal of the olfactory bulbs vs. genetic modulation).

## Introduction

At present, depression is one of the most common psychiatric disorders, but its pharmacotherapy and pathophysiology are still unclear. The etiology of depression is multifactorial and related to disturbed monoaminergic transmission, changes in receptor function, dysregulation of the stress axis, a reduction of neurogenesis, activated inflammatory processes, or increased oxidative stress (Moniczewski et al. 2015; Smaga et al. 2015). Recent observations strongly support the involvement of the endocannabinoid (eCB) system in the pathogenesis of depression. In preclinical studies, genetic deletion of the cannabinoid (CB_1_) receptor or the CB_1_ receptor blockade resulted in depressive-like behavior (Sanchis-Segura et al. 2004; Aso et al. 2008; Steiner et al. 2008; Mikics et al. 2009). On the other hand, the facilitation of eCB signaling exerts antidepressant-like behavioral responses in rodents, which were observed after: anandamide (AEA) administration (Umathe et al. 2011), AEA uptake inhibition (Hill and Gorzalka 2005; Adamczyk et al. 2008; Mannucci et al. 2011; Umathe et al. 2011), eCB degrading enzyme inhibition (Gobbi et al. 2005; Adamczyk et al. 2008), or by direct CB_1_ agonists (Bambico et al. 2007; Haring et al. 2013; Smaga et al. 2014b). Moreover, reduction of eCB signaling was linked to depressive-like behavior in several models of depression (Hill et al. 2005, 2008a; Eisenstein et al. 2010; Dubreucq et al. 2012; Vinod et al. 2012). In a human study, administration of a CB_1_ antagonist (rimonabant) (Christensen et al. 2007; Horder et al. 2009) for treatment of obesity resulted in the development of depression and anhedonia. Additionally, augmentation of eCB signaling evoked different changes implicated in antidepressant effects, such as modulation of neurotransmitter release, regulation of the stress axis, promotion of neurogenesis, and normalization of the excitation state (Smaga et al. 2014a, b).

The aim of the present study was to perform a complex analysis to the eCB system (i.e., endogenous ligands, enzymes involved in eCB metabolism, and CB receptors) in the pathogenesis of depression by using valid animal models of depression: Wistar Kyoto (WKY) rats and bulbectomized (OBX) animals. We focused on determining whether the levels of eCBs (i.e., AEA and 2-arachidonoylglycerol (2-AG)), the expression of the eCB synthesizing enzymes (N-acyl phosphatidylethanolamine phospholipase D (NAPE-PLD) and diacylglycerol lipase α (DAGLα)), or eCB degrading enzymes (fatty acid amide hydrolase (FAAH) and monoacylglycerol lipase (MAGL)) were altered in particular models of depression. Finally, we measured the expression of CB receptors and CB_1_ receptor density. The olfactory bulbectomy is used as a model of depression induced by an injury and is characterized by all of the proper criteria of validity (face, construct, and predictive) that demonstrate a good clinical picture of depression (Abelaira et al. 2013). On the other hand, WKY rats are an example of a genetic model of depression and are characterized by hyperactive responses to stress with dysregulation of the hypothalamic-pituitary-adrenal (HPA) axis. Both of these models demonstrated behavioral, biochemical, and physiological abnormalities reminiscent of depressive-like symptom (Will et al. 2003; Yuan and Slotnick 2014).

## Experimental Procedures

### Animals

The experiments were performed on male Wistar and WKY rats (Charles River, Germany) weighing 280–300 g (approximately 8–9 weeks old). The animals were kept on a normal day-night cycle at 22 ± 2 °C with access to food and water ad libitum. All experiments were carried out in accordance with the EU directive 2010/63/EU and with approval of the Bioethics Commission as compliant with the Polish Law (21 August 1997). There were 6–8 animals per group.

#### Olfactory Bulbectomy

During anesthesia (ketamine hydrochloride and xylazine mixture in 3:1 *v*/*v*, im), bilateral olfactory bulbectomy was performed on Wistar rats. Metoxicam (0.05 mg/kg, sc) was given as an analgesic and anti-inflammatory drug 60 min before the surgery and for 2 days after the surgery. Two burr holes were drilled on either side of the skull (taking care not to damage the prefrontal cortex), 2 mm in diameter, 8 mm anterior to the bregma, and 2 mm from the midline of the frontal bone overlying the olfactory bulbs (coordinates were taken from the Rat Brain Atlas, Paxinos and Watson 1998). The olfactory bulbs were removed by suction, and the burr holes were filled with a hemostatic sponge to control bleeding. The skin was closed. Animals receiving sham (SHAM) surgery underwent a similar procedure but did not have their olfactory bulbs removed. During the 14-day recovery, the animals were handled daily by the experimenter to eliminate any aggressiveness that would otherwise develop (Leonard and Tuite 1981; Smaga et al. 2012). After the behavioral procedures, the OBX rats were sacrificed through decapitation, and their brains were rapidly removed and examined for signs of cortical damage or incomplete removal of the olfactory bulbs. Such animals were excluded from the data analysis.

### Behavioral Tests

#### Locomotor Activity

Open field locomotor activity was recorded individually for each animal in Opto-Varimex cages (Columbus Instruments, Columbus, OH, USA) linked on-line to a compatible IBM PC. Each cage (43 × 44 cm) was equipped with 15 infrared emitters located on the *x* and *y* axes, 3 cm from the floor and with the same number of receivers on the opposite walls of the cage. The rats’ behavior was analyzed using Auto-Track software (Columbus Instruments, Columbus, OH, USA). Locomotor activity was defined as a breakage of three consecutive photobeams. Locomotor activity was defined as horizontal activity and presented as distance traveled in centimeter during 30-min trial (with 5-min intervals).

#### Forced Swimming Test (FST)

On the first day of the forced swim (i.e., pre-test), the rats were placed individually in a cylinder (50 cm high × 23 cm in diameter) filled to a 30-cm depth with water (25 ± 1 °C) for 15 min; they were then removed from the water, dried with towels, placed in a warmer enclosure for 15 min, and were then returned to their home cages, as described previously (Frankowska et al. 2010). The cylinders were emptied and cleaned between rats. Twenty-four hours following the first exposure, the rats were tested for 5 min (300 s) under identical conditions. Test sessions were scored by two observers unaware of the treatment condition, and the immobility time was measured. A rat was considered to be immobile if it was only making movements necessary to keep its head above water.

### Tissue Isolation

For biochemical analysis, experimental-naïve rats (WKY, OBX, and corresponding controls) were sacrificed through decapitation, and their brains were rapidly removed. Selected brain structures (i.e., the prefrontal cortex, frontal cortex, hippocampus, dorsal striatum, nucleus accumbens, and cerebellum) were isolated using brain matrix according to The Rat Brain Atlas (Paxinos and Watson 1998), immediately frozen on dry ice and stored at −80 °C for liquid chromatography tandem mass spectrometry (LC-MS/MS) and Western blot analysis. Separate groups of animals were used for autoradiography; their brains were rapidly dissected, frozen by dry-ice bath using heptane, and were later stored at −80 °C until sectioned.

### Biochemical Analysis

#### LC-MS/MS

##### Reagents

All chemical solvents and standards were of analytical grade. Standards of AEA and 2-AG were obtained from Tocris (Bristol, United Kingdom), AEA-d_4_ and 2-AG-d_5_ from Cayman Chemical (USA), acetonitrile and chloroform from Merck (Darmstadt, Germany), and methanol and formic acid from POCh (Katowice, Poland). Standard stock solutions were prepared in ethanol (AEA and AEA-d_4_) or acetonitrile (2-AG and 2-AG-d_5_). All stock solutions were stored at −80 °C. Further dilutions were carried out appropriately in acetonitrile.

##### Lipid Extraction from Brain Tissue

The brain tissues were weighed and subjected to eCB extraction. Extraction was carried out by the modified methods of isolation of lipid compounds, as described previously (Bystrowska et al. 2014b). Tissues were homogenized using a sonicator (UP50H, Hielscher) in the ice-cold mixture of methanol and chloroform (1:2, *v*/*v*) in the proportion of 10 mg of wet tissue to 150 μl of solvent to quench any possible enzymatic reaction that may interfere with the analysis. Next, 150 μl of homogenate were mixed with 2 μl of internal standard (AEA-d_4_, concentration 10 μg/ml; 2-AG-d_5_, concentration 100 μg/ml), 250 μl of formic acid (pH 3.0; 0.2 M), and 1500 μl of extraction mixture (methanol:chloroform 1:2, *v*/*v*). The internal standard indicates analyte loss during sample work-up. Afterward, samples were vortexed for 30 s and centrifuged for 10 min at 2000 rpm. Organic phases were collected and dried under a stream of nitrogen at 40 °C. The residue was dissolved in 40 μl of acetonitrile, and 10 μl of the reconstituted extract was injected into the LC-MS/MS system for quantitative analysis.

##### LC-MS/MS Conditions

LC was performed using an Agilent 1100 (Agilent Technologies, Waldbronn, Germany) LC system. Chromatographic separation was carried out with a Thermo Scientific BDS HYPERSIL C18 column (100 × 3 mm I.D., 3-μm particle size). The advance column with precolumn (10 × 3 mm I.D., 3-μm particle size) was set at 40 °C with a mobile phase flow rate of 0.3 ml/min. Gradient elution mobile phases consisted of formic acid (0.02 M) in water (solvent A) and formic acid (0.02 M) in acetonitrile (solvent B). The gradient began initially at 0% A during 1 min, increasing linearly to 90% at 2 min; this was maintained for 2 min and then was decreased to 0% at 6 min. Finally, the last 4 min of the analysis remained at 100% B. Sample temperature was maintained at 4 °C in the autosampler prior to analysis. A sample volume of 10 μl was injected into the analytical column for compound analysis.

MS/MS analyses were accomplished on an Applied Biosystems MDS Sciex (Concord, Ontario, Canada) API 2000 triple quadrupole mass spectrometer equipped with an electrospray ionization (ESI) interface. ESI was performed in the positive ionization mode. A standard polypropylene glycol solution (PPG standard) was used for instrument tuning and mass calibration at unit mass resolution according to the Applied Biosystems manual. The mass spectrometer was operated with a dwell time of 200 ms. To find the optimal parameters of ion path and ion source of the studied compound, the quantitative optimization was done by direct infusion of standards using a Hamilton syringe pump (Hamilton, Reno, NV, USA). Multiple reaction monitoring (MRM) mode of the dominant product ion for each eCB was realized using the optimal conditions. The ion source parameters were as follows: ion spray voltage (IS)—5500 V, nebulizer gas (gas 1)—30 psi, turbo gas (gas 2)—10 psi, temperature of the heated nebulizer (TEM)—400 °C, and curtain gas (CUR)—25 psi. Comparison of pair ions (precursor and product ion *m*/*z* values) and LC retention times with standards served to confirm the identification of eCB in the samples investigated. Ion pairs were 348/62 for AEA, 379/287 for 2-AG, 352/66 for AEA-d_4_, and 384/292 for 2-AG-d_5_. Data acquisition and processing were accomplished using the Applied Biosystems Analyst version 1.4.2 software.

##### Calibration Curve and Quantification

The concentrations of eCBs in the samples were calculated using the calibration curve that was prepared on the same day and analyzed in the same analytical run. Calibration curves were constructed after analysis of the samples of brain tissues collected from naïve rats. The homogenates were spiked with AEA to the following concentrations: blank, 0.1, 1, 10, 25, 50, and 100 ng/g. For 2-AG, the following solutions were used: blank, 0.4, 1, 5, 10, 25, and 50 μg/g. AEA-d_4_ and 2-AG-d_5_ were used as the internal standards. The samples were analyzed according to the procedure described above (“Lipid Extraction from Brain Tissue” section).

#### Western Blot

Frozen brain structures were homogenized in buffer to the homogenization (1 mM HEPES, 0.1 M DTT, 0.1 mM EGTA (pH 7.2–7.8), COMPLETE, and sterile water) using a homogenizer ball (Bioprep-24, Allsheng, China) (10 s at 10,000 rpm) and were then denatured for 2 min at 85 °C, 2 min in ice, 5 min at 85 °C, and finally 2 min in ice. For protein determination, a bicinchoninic acid assay (BCA) protein assay kit (Serva, Germany) was used. Protein samples (30 μg) were resolved by 10% SDS polyacrylamide gels and transferred to a polyvinylidene difluoride (PVDF) membrane. Membranes were blocked in 3% non-fat dry milk, and separate sets of membranes were probed with rabbit anti-CB_1_ monoclonal antibody (1:800; ab172970; Abcam, UK; Kaczocha et al. 2015), rabbit anti-CB_2_ polyclonal antibody (1:500; 101550; Cayman, USA; Álvaro-Bartolomé and García-Sevilla 2013), goat anti-NAPE-PLD polyclonal antibody (1:200; ab95397; Abcam, UK; Kaczocha et al. 2015), goat anti-MAGL polyclonal antibody (1:200; ab24701; Abcam, UK), rabbit anti-DAGLα polyclonal antibody (1:200; ab81984; Abcam, UK), and mouse anti-FAAH monoclonal antibody (1:200; sc-100739; Santa Cruz Biotechnology, USA). The expressions of CB_1_, CB_2_, NAPE-PLD, FAAH, DAGLα, or MAGL were evaluated relative to that of glyceraldehyde 3-phosphate dehydrogenase (GAPDH) using rabbit anti-GAPDH polyclonal antibody (1:1500, sc-25778, Santa Cruz Biotechnology, USA). Blots were washed and incubated with donkey anti-goat secondary antibody (1:6000; 926-68074; Li-cor, USA), goat anti-rabbit secondary antibody (1:6000; 926-68071; Li-cor, USA), or goat anti-mouse (1:6000; 926-32210; Li-cor, USA) and visualized with a fluorescence detection Odyssey Clx (Li-cor, USA). Analysis was performed using Image Studio v.2.1. All data were expressed as % of control.

#### Autoradiography

Consecutive coronal sections (12 μm) of rat brains were cut on a cryostat microtome (Leica CM 1850, Germany) at −22 ± 2 °C and were thaw-mounted on polylysine-coated slides. Five coronal sections were mounted on a single slide and stored at −80 °C. Receptor binding assay with [^3^H]CP55,940 (PerkinElmer, USA; specific activity 164.5 Ci/mmol) was performed using the procedure described previously with some modifications (Adamczyk et al. 2012). The slices were preincubated for 2 h at 37 °C in buffer (containing 50 mM Tris–HCl with 5% bovine serum albumin; pH 7.5).

Total binding was measured by incubating the appropriate tissue sections with [^3^H]CP55,940 (2 nM) for 2 h at 37 °C in the buffer (see above). To determine the non-specific binding, parallel sections were incubated additionally in the presence of 10 μM CP55,940 (Sigma-Aldrich, USA). Following the incubation period, tissue sections were washed twice in an ice-cold (4 °C) buffer (containing 50 mM Tris–HCl with 1% bovine serum albumin; pH 7.5) for 2 h and twice in distilled water (4 °C) for 1 min, and then dried with cool air.

Radiolabeled, dried tissue sections were exposed to tritium-sensitive screens (3H-Fujifilm imaging plates; Bas-TR2025, Fuji Photo Film, Japan) for 10 days at 4 °C. Induced autoradiograms were read out with a reader (BAS-5000 IP Image Reader v.1.1, Fujifilm), and quantitative analysis was performed using the Fujifilm software (IMAGE GAUGE, v.4.0.). Optical densities of gray values on the film were converted into bound radioactivity with a polynomial regression curve derived from autoradiographic [^3^H]microscales (RPA 510, Amersham, UK) used as calibration markers. Data (fmol/mg tissue) are expressed as the mean of the percentage of the control levels ± standard error of mean (SEM).The binding signal was analyzed in several brain areas, which were identified by comparing autoradiographic images with appropriate figures from The Rat Brain Atlas of Paxinos and Watson (1998) (Table 1).

**Table 1:**
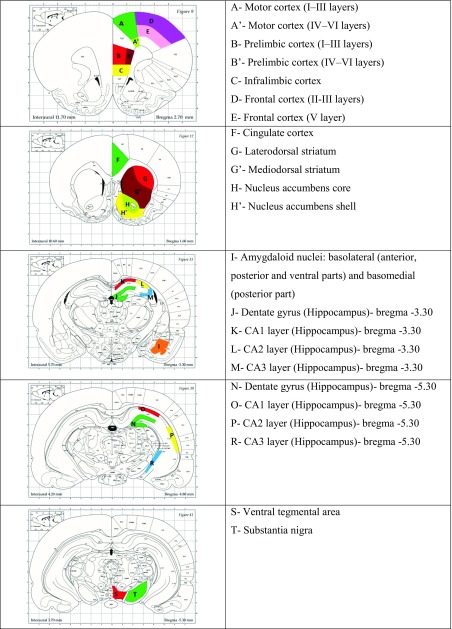
The obtain regions used for quantitative analysis, chosen according to The Rat Brain Atlas (Paxinos and Watson 1998). The color outlines show the brain areas in which optical densities were quantified

### Statistical Analyses

All data were expressed as the mean ± SEM. Statistical analyses were performed with Student’s *t* test. *P* < 0.05 was considered statistically significant.

## Results

### Behavioral Tests

#### FST

The OBX rats displayed longer (by 30.7%) (*t* = 3.652; df = 14; *p* = 0.0026) immobility time compared with the SHAM controls (OBX rats 140.5 ± 8.0; SHAM-operated rats 107.5 ± 4.1). The WKY rats demonstrated a significantly increased (by 45.2%) (*t* = 2.614; df = 14; *p* = 0.0204) immobility time compared with the control group (WKY rats 126.9 ± 10.3; Wistar rats 87.4 ± 11.1).

#### Locomotor Activity

Removal of the olfactory bulbs evoked a significant enhancement of rat locomotor activity during 5-min (OBX rats 1714 ± 125; SHAM-operated rats 1132 ± 80; *t* = 3.929; df = 14; *p* = 0.015) and 30-min (OBX rats 5042 ± 330; SHAM-operated rats 3282 ± 333; *t* = 3.753; df = 14; *p* = 0.0021) trials. The locomotor activity of the WKY rats was unchanged compared with that of the control animals in the 5-min (WKY rats 1085 ± 164; Wistar rats 1025 ± 78) or 30-min (WKY rats 2883 ± 48; Wistar rats 2468 ± 209) trials.

### Biochemical Analyses

#### eCB Tissue Content

##### AEA

**Fig. 1:**
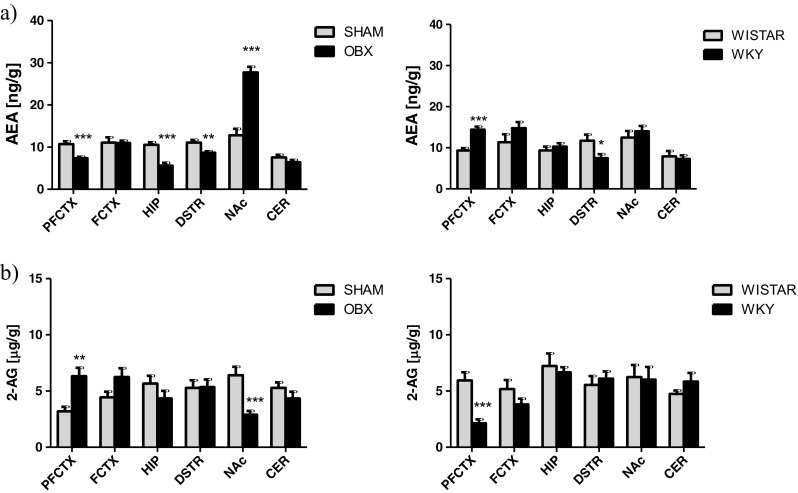
Changes in the eCBs levels; AEA (**a**) and 2-AG (**b**) in brain structures from OBX and WKY rats. *AEA* anandamide, *2-AG* 2-arachidonoylglycerol, *PFCTX* prefrontal cortex, *FCTX* frontal cortex, *HIP* hippocampus, *DSTR* dorsal striatum, *NAc* nucleus accumbens, *CER* cerebellum, *OBX* bulbectomized rats, *WKY* Wistar Kyoto rats. All data are expressed as mean ± SEM. *N* = 8 rats/group. *p < 0.05; **p < 0.01; ***p < 0.001 vs SHAM-operated or Wistar rats

**Fig. 2:**
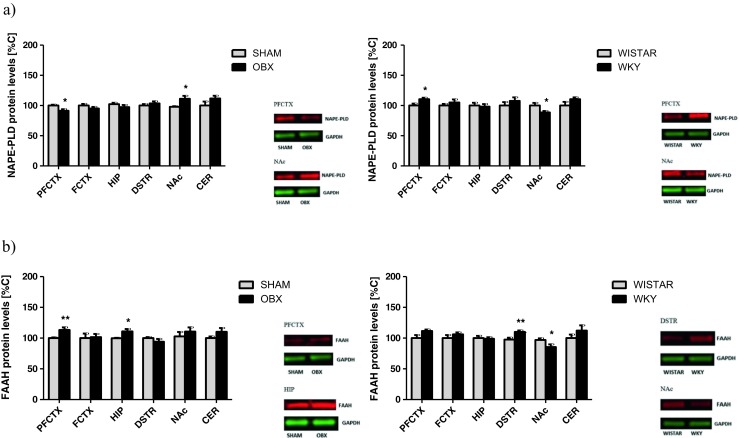
Changes in the expression of metabolizing enzymes of AEA synthetizing enzyme—NAPE-PLD (**a**) and degrading enzyme—FAAH (**b**) in brain structures from OBX and WKY rats. *AEA* anandamide, *NAPE*-*PLD* N-acyl phosphatidylethanolamine phospholipase D, *FAAH* fatty acid amide hydrolase, *PFCTX* prefrontal cortex, *FCTX* frontal cortex, *HIP* hippocampus, *DSTR* dorsal striatum, *NAc* nucleus accumbens, *CER* cerebellum, *OBX* bulbectomized rats, *WKY* Wistar Kyoto rats. All data are expressed as mean ± SEM. *N* = 8 rats/group. *p < 0.05; **p < 0.01 vs SHAM-operated or Wistar rats

When comparing the OBX to the SHAM rats (Fig. 2a), we found a significant reduction of the AEA levels in the prefrontal cortex (*t* = 4.487; df = 14; *p* = 0.0005), hippocampus (*t* = 5.405; df = 14; *p* < 0.0001), and dorsal striatum (*t* = 3.328; df = 14; *p* = 0.005). At the same time, removal of the olfactory bulbs evoked a significant enhancement of the AEA levels in the nucleus accumbens (*t* = 7.394; df = 14; *p* < 0.0001) (Fig. 1a).

In the WKY rats, the levels of AEA either increased in the prefrontal cortex (*t* = 5.499; df = 14; *p* < 0.0001) or decreased in the dorsal striatum (*t* = 2.384; df = 14; *p* = 0.0318), while no changes in the rest of the brain structures were observed (Fig. 1a).

##### 2-AG

The 2-AG levels either increased in the prefrontal cortex (*t* = 3.683; df = 14; *p* = 0.0025) or decreased in the nucleus accumbens (*t* = 4.316; df = 14; *p* = 0.0007) in the OBX rats compared with those in the SHAM-operated controls (Fig. 1b).

In the WKY rats, a decrease in 2-AG levels was observed in the prefrontal cortex (*t* = 4.715; df = 14; *p* = 0.0003) (Fig. 1b).

#### Enzyme Protein Expression

##### NAPE-PLD and FAAH

Removal of the olfactory bulbs induced either a decrease in NAPE-PLD protein expression in the prefrontal cortex (*t* = 3.001; df = 14; *p* = 0.0111) or an increase in the expression of this protein in the nucleus accumbens (*t* = 2.754; df = 14; *p* = 0.0175) compared with SHAM-operated rats (Fig. 2a). In the WKY rats, an increase in NAPE-PLD protein expression was observed in the prefrontal cortex (*t* = 2.722; df = 14; *p* = 0.0185), and a decrease of this protein expression was shown in the nucleus accumbens (*t* = 2.266; df = 14; *p* = 0.0427) (Fig. 2a).

In the OBX rats, an increase of FAAH protein expression was seen in the prefrontal cortex (*t* = 3.058; df = 14; *p* = 0.0099) and hippocampus (*t* = 2.887; df = 14; *p* = 0.0137) (Fig. 2b). In the WKY rats, either an increase in FAAH protein expression in the dorsal striatum (*t* = 3.209; df = 14; *p* = 0.0075) or a decrease in this protein expression in the nucleus accumbens (*t* = 2.255; df = 14; *p* = 0.0436) was noted (Fig. 2b).

##### DAGLα and MAGL

**Fig. 3:**
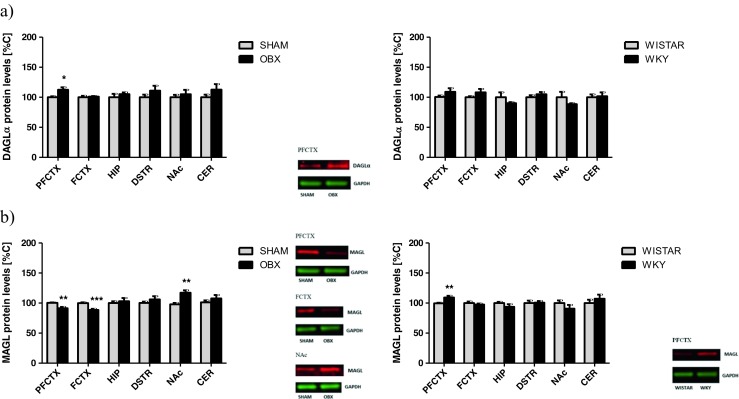
Changes in the expression of metabolizing enzymes of 2-AG synthetizing enzyme—DAGLα (**a**) and degrading enzyme—MAGL (**b**) in brain structures from OBX and WKY rats. *2*-*AG* 2-arachidonoylglycerol, *DAGLα* diacylglycerol lipase α, *MAGL* monoacylglycerol lipase, *PFCTX* prefrontal cortex, *FCTX* frontal cortex, *HIP* hippocampus, *DSTR* dorsal striatum, *NAc* nucleus accumbens, *CER* cerebellum, *OBX* bulbectomized rats, *WKY* Wistar Kyoto rats. All data are expressed as mean ± SEM. *N* = 8 rats/group. *p < 0.05; **p < 0.01; ***p < 0.001 vs SHAM-operated or Wistar rats

In the OBX rats, an increase of DAGLα protein expression was noted in the prefrontal cortex (*t* = 2.919; df = 14; *p* = 0.0129) compared with that in the SHAM-operated rats, while in the WKY rats, the levels of DAGLα protein expression did not change (Fig. 3a).

Removal of the olfactory bulbs in rats evoked a reduction of MAGL protein expression in the prefrontal cortex (*t* = 3.876; df = 14; *p* = 0.0022) and frontal cortex (*t* = 4.994; df = 14; *p* = 0.0003), while in the nucleus accumbens, higher levels of MAGL protein expression were noted (*t* = 4.151; df = 14; *p* = 0.0013) (Fig. 3b). In the WKY rats, only a significant increase of MAGL protein expression was seen in the prefrontal cortex (*t* = 3.073; df = 14; *p* = 0.0097) (Fig. 3b).

#### CB_1_ Receptor Autoradiography

Differentiation of radioligand binding to the CB_1_ receptors was dependent on the structure and was in the range of 40.40 ± 2.18 (frontal cortex layer II/III) to 384.10 ± 16.38 (substantia nigra) fmol/mg tissue in the control animals. Low values of radioligand binding (40–60 fmol/mg tissue) were seen in the motor cortex, prelimbic cortex, infralimbic cortex, frontal cortex, nucleus accumbens, amygdala, and ventral tegmental area; the mean values (between 70 and 110 fmol/mg tissue) were observed in the dorsal striatum and hippocampal areas; and the maximum binding (greater than 350 fmol/mg tissue) was observed in the substantia nigra.

In the OBX rats, the binding of [^3^H]CP55,940 either increased in the prelimbic cortex (IV–VI layers) (*t* = 4.031; df = 10; *p* = 0.0024) or decreased in the laterodorsal striatum (*t* = 2.6; df = 10; *p* = 0.0265) compared with that in the SHAM-operated rats (Table 2). The binding of [^3^H]CP55,940 in the WKY rats increased in the motor cortex (I–III layers) (*t* = 3.399; df = 10; *p* = 0.0068), motor cortex (IV–VI layers) (*t* = 2.558; df = 10; *p* = 0.0285), prelimbic cortex (I–III layers) (*t* = 5.07; df = 10; *p* = 0.0005), prelimbic cortex (IV–VI layers) (*t* = 4.593; df = 10; *p* = 0.001), infralimbic cortex (*t* = 5.591; df = 10; *p* = 0.0002), frontal cortex (II–III layers) (*t* = 4.967; df = 10; *p* = 0.0006), frontal cortex (V layer) (*t* = 5.258; df = 10; *p* = 0.0004), cingulate cortex (*t* = 3.146; df = 10; *p* = 0.0104), laterodorsal striatum (*t* = 2.528; df = 10; *p* = 0.03), nucleus accumbens core (*t* = 3.028; df = 10; *p* = 0.0127), nucleus accumbens shell (*t* = 2.706; df = 10; *p* = 0.0221), amygdala (*t* = 2.63; df = 10; *p* = 0.0252), dentate gyrus of the hippocampus (*t* = 2.999; df = 10; *p* = 0.0134 and *t* = 4.475; df = 10; *p* = 0.0012), and the hippocampal CA1 (*t* = 2.943; df = 10; *p* = 0.0147) and CA3 layers (*t* = 3517; df = 10; *p* = 0,0056) compared with that in the Wistar rats (Table 3).

**Table 2:** The binding of [^3^H]CP55.940 to CB_1_ receptors in various brain areas in OBX rats

	SHAM	OBX
Motor cortex (I–III layers)	100.0 ± 7.97	119.9 ± 8.08
Motor cortex (IV–VI layers)	100.0 ± 7.64	101.3 ± 5.68
Prelimbic cortex (I–III layers)	100.0 ± 10.56	92.1 ± 5.11
Prelimbic cortex (IV–VI layers)	100.0 ± 5.08	143.9 ± 9.63**
Infralimbic cortex	100.0 ± 10.10	95.8 ± 5.22
Frontal cortex (II–III layers)	100.0 ± 8.45	109.6 ± 4.49
Frontal cortex (V layer)	100.0 ± 12.07	101.3 ± 5.87
Cingulate cortex	100.0 ± 6.28	111.8 ± 5.20
Laterodorsal striatum	100.0 ± 5.93	82.1 ± 3.53*
Mediodorsal striatum	100.0 ± 4.80	103.1 ± 6.29
Nucleus accumbens core	100.0 ± 10.65	100.1 ± 9.58
Nucleus accumbens shell	100.0 ± 13.75	89.9 ± 9.20
Amygdala	100.0 ± 7.98	102.7 ± 5.38
Dentate gyrus (hippocampus)—bregma—3.30	100.0 ± 4.15	112.6 ± 6.53
CA1 layer (hippocampus)—bregma—3.30	100.0 ± 6.15	93.9 ± 7.20
CA2 layer (hippocampus)—bregma—3.30	100.0 ± 12.66	121.3 ± 13.64
CA3 layer (hippocampus)—bregma—3.30	100.0 ± 4.75	101.5 ± 11.83
Dentate gyrus (hippocampus)–bregma—5.30	100.0 ± 6.67	95.0 ± 1.71
CA1 layer (hippocampus)—bregma—5.30	100.0 ± 10.85	114.1 ± 4.68
CA2 layer (hippocampus)—bregma—5.30	100.0 ± 8.78	112.0 ± 10.76
CA3 layer (hippocampus)—bregma—5.30	100.0 ± 7.15	94.3 ± 7.37
Ventral tegmental area	100.0 ± 4.85	97.1 ± 8.15
Substantia nigra	100.0 ± 4.86	99.9 ± 5.95

The number of animals in all examined groups was *n* = 6. Data are presented as the mean percent binding (±S.E.M.) relative to control (SHAM)

*OBX* bulbectomized rats, *SHAM* SHAM-operated rats

*p < 0.05; **p < 0.01 vs. SHAM

**Table 3:** The binding of [^3^H]CP55.940 to CB_1_ receptors in various brain areas in WKY rats

	WISTAR	WKY
Motor cortex (I–III layers)	100.0 ± 3.37	117.4 ± 3.86**
Motor cortex (IV–VI layers)	100.0 ± 5.89	115.7 ± 1.78*
Prelimbic cortex (I–III layers)	100.0 ± 1.66	129.9 ± 5.66***
Prelimbic cortex (IV–VI layers)	100.0 ± 5.73	135.2 ± 5.08***
Infralimbic cortex	100.0 ± 2.36	123.5 ± 3.48***
Frontal cortex (II–III layers)	100.0 ± 7.61	138.5 ± 1.48***
Frontal cortex (V layer)	100.0 ± 7.45	148.5 ± 5.43***
Cingulate cortex	100.0 ± 7.23	128.9 ± 5.66*
Laterodorsal striatum	100.0 ± 6.72	120.5 ± 4.54*
Mediodorsal striatum	100.0 ± 4.96	110.5 ± 3.31
Nucleus accumbens core	100.0 ± 7.90	129.7 ± 5.83*
Nucleus accumbens shell	100.0 ± 5.67	126.2 ± 7.82*
Amygdala	100.0 ± 8.04	123.2 ± 3.66*
Dentate gyrus (hippocampus)—bregma—3.30	100.0 ± 4.73	128.8 ± 8.35*
CA1 layer (hippocampus)—bregma—3.30	100.0 ± 4.71	113.2 ± 5.69
CA2 layer (hippocampus)—bregma—3.30	100.0 ± 4.54	117.0 ± 8.53
CA3 layer (hippocampus)—bregma—3.30	100.0 ± 5.54	103.4 ± 9.02
Dentate gyrus (hippocampus)—bregma—5.30	100.0 ± 6.08	129.0 ± 2.21**
CA1 layer (hippocampus)—bregma—5.30	100.0 ± 4.52	135.6 ± 11.24*
CA2 layer (hippocampus)—bregma—5.30	100.0 ± 8.55	114.7 ± 11.53
CA3 layer (hippocampus)—bregma—5.30	100.0 ± 5.80	123.3 ± 3.18**
Ventral tegmental area	100.0 ± 13.07	124.9 ± 14.88
Substantia nigra	100.0 ± 8.00	100.7 ± 5.72

The number of animals in all examined groups was *n* = 6. Data are presented as the mean percent binding (±S.E.M.) relative to control (Wistar)

*WKY* Wistar Kyoto rats

*p < 0.05; **p < 0.01; ***p < 0.001 vs. SHAM

#### CB_1_ and CB_2_ Receptor Expression

**Fig. 4:**
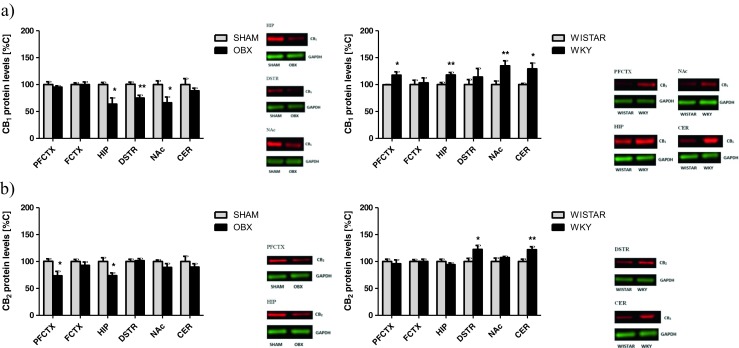
Changes in the expression of CB receptors CB_1_ (**a**) and CB_2_ (**b**) in brain structures from OBX and WKY rats. *PFCTX* prefrontal cortex, *FCTX* frontal cortex, *HIP* hippocampus, *DSTR* dorsal striatum, *NAc* nucleus accumbens, *CER* cerebellum, *OBX* bulbectomized rats, *WKY* Wistar Kyoto rats. All data are expressed as mean ± SEM. *N* = 8 rats/group. *p < 0.05; **p < 0.01 vs SHAM-operated or Wistar rats

Removal of the olfactory bulbs evoked a decrease in CB_1_ receptor expression in the hippocampus (*t* = 2.994; df = 14; *p* = 0.0112), dorsal striatum (*t* = 3.908; df = 14; *p* = 0.0021), and nucleus accumbens (*t* = 2.648; df = 14; *p* = 0.0212) (Fig. 4a). In the WKY rats, an increase of CB_1_ receptor expression was observed in the prefrontal cortex (*t* = 2.837; df = 14; *p* = 0.015), hippocampus (*t* = 3.076; df = 14; *p* = 0.0096), nucleus accumbens (*t* = 3.055; df = 14; *p* = 0.01), and cerebellum (*t* = 2.692; df = 14; *p* = 0.0196) (Fig. 4a).

In the OBX rats, a decrease of CB_2_ receptor expression was noted in the prefrontal cortex (*t* = 2.857; df = 14; *p* = 0.0144) and hippocampus (*t* = 2.988; df = 14; *p* = 0.0113), while in the WKY rats, an increase of CB_2_ receptor expression was present in the dorsal striatum (*t* = 2.396; df = 14; *p* = 0.0338) and cerebellum (*t* = 3.13; df = 14; *p* = 0.0087) (Fig. 4b).

## Discussion

The present results show that behavioral depressive-like behavior manifested by the increased immobility time in the FST (without reduction in the locomotor activity) is paralleled by several alterations in the eCB system components. We used two models of depression utilizing completely different mechanisms for inducing a depressive phenotype. Removal of the olfactory bulbs evokes behavioral, neurochemical, and immunological changes, which are similar to alterations observed in depressive patients (Kelly et al. 1997; Song and Leonard 2005; Wang et al. 2010; Jastrzebska et al. 2015) and are reversed by chronic treatment with antidepressants (Smaga et al. 2012; Yuan and Slotnick 2014). The other animal model of depression, WKY rats, exhibits depressive-like behavior in the FST (Pare 1994; Armario et al. 1995; Lahmame and Armario 1996; Rittenhouse et al. 2002) and open field test (Pare 1994; Berton et al. 1997), together with abnormalities in some central neurochemical-dopaminergic (Jiao et al. 2006) and noradrenergic (Pardon et al. 2002) systems and hormonal dysregulation of the HPA (Solberg et al. 2001), as well as lower levels of brain-derived neurotrophic factor (BDNF) in the CA3 region of the hippocampus (Malkesman and Weller 2009).

In our neurochemical analysis, the OBX rats showed reduced AEA levels and increased 2-AG levels in the prefrontal cortex, while in the WKY rats, the cortical AEA levels were increased and the 2-AG contents were significantly reduced. The above data in the particular animal models of depression are partly supported by human and animal studies in which prefrontal eCB levels were either increased in alcoholic suicide victims (Vinod et al. 2005) and in depressed suicide victims (Hungund et al. 2004) or reduced following exposure to chronic unpredictable stress (CUS) (Hill et al. 2008a). Additionally, clinical trials of untreated depressed patients demonstrated changes in serum eCB levels that parallel the changes seen in the WKY rats in our study. In fact, an increase in the AEA level (Hill et al. 2008c; Ho et al. 2012) and a decrease in the 2-AG level were observed in human female patients with major depression (Hill et al. 2008c). It should be noted that the WKY rats are a useful model that captures specific functional domains relevant to clinical depression and would seem most consistent with a melancholic depression profile showing severe symptoms of anxiety and depression (Willner and Belzung 2015). The WKY rats seem to be a genetic model of childhood depression (Malkesman and Weller 2009), while the OBX rats are more representative for depression in adults.

Changes in the levels of eCBs observed in the prefrontal cortex reflect the alteration in the expression of enzymes involved in the metabolism of these compounds. Thus, an increase in the levels of AEA in the WKY rats can result from an increase in the expression of NAPE-PLD, which is the AEA synthesizing enzyme, while a decrease in 2-AG levels in this structure may be evoked by higher expression of MAGL, a 2-AG degrading enzyme. Similar correlations were demonstrated in the OBX rats, where the reduction of AEA levels might result from the reduced synthesis by NAPE-PLD or enhanced degradation by FAAH. The increase in DAGLα expression (enzyme synthesizing 2-AG) and the decrease in MAGL expression probably caused the increase in the levels of 2-AG in the prefrontal cortex of the OBX rats. The above findings for the first time show the importance of alterations in eCB metabolizing enzymes as a potential factor inducing changes in the levels of eCBs in animal models of depression. These data complement only one report in which the FAAH activity was studied in the prefrontal cortex in rats subjected to the CUS procedure (Hill et al. 2008a).

The increased AEA levels in the prefrontal cortex of the WKY rats may be involved in reduction of 5-HTergic neurotransmission and in depression-like behavior. As shown in these animals, increases in the AEA levels via activation of CB_1_ receptors up-regulated 5-HT_2A_ receptor activity while concurrently down-regulating 5-HT_1A_ receptor activity (Hill et al. 2006). The latter neuroadaptations were observed in depressed patients (Drevets et al. 1999; Bhagwagar et al. 2004). The rise of the AEA levels may also induce emotional discomfort during depression and reinforce emotional memories of aversive stimuli (Morena et al. 2014). The activation of the eCB system via CB_1_ receptors strongly modulates memory consolidation (Lafourcade et al. 2007). The latter changes may be further enhanced by the increased CB_1_ receptor density in various brain areas in the WKY rats, such as in the cortical areas, which was demonstrated in our study. Furthermore, the increased expression of CB_1_ receptors in the prefrontal cortex, seen in our study in the WKY rats and in other various animal models of depression induced by stress factors (Bortolato et al. 2007; Hill et al. 2008a; McLaughlin et al. 2013), seems to be a common characteristic change for this type of symptom. The elevated AEA levels also decreased the concentration, metabolism, and physiological functions of 2-AG via vanilloid receptors (TRPV1) (Maccarrone et al. 2008; Bystrowska et al. 2014a), and reduced 2-AG levels were seen in the prefrontal cortex in the WKY rats in our study. To partly support the 2-AG significance in the prefrontal cortex, the antidepressant drug tranylcypromine, contrary to the WKY rats, increased the 2-AG levels in the prefrontal cortex (Hill et al. 2008b).

On the other hand, reduction of AEA levels in the prefrontal cortex in the present study following bulbectomy might be associated with alterations in monoaminergic signaling. In fact, in the OBX rats, the extracellular levels of dopamine (Prins et al. 2010), 5-HT (van der Stelt et al. 2005; Jastrzebska et al. 2015), and noradrenaline (Jastrzebska et al. 2015) were reduced in the prefrontal cortex. Homeostasis of eCB signaling may remain stable due to up-regulation of the 2-AG levels (seen in this study) or/and of the CB_1_ receptor density in the prefrontal cortex (Rodriguez-Gaztelumendi et al. 2009). The increased CB_1_ receptor density was also confirmed in our study, although this increase was demonstrated only in the prelimbic cortex (IV–VI layers) using autoradiography, while labeling of proteins with specific antibodies did not show changes in CB_1_ receptors, and even a decrease in the CB_2_ receptor expression in the OBX rats was observed. The reason for the differences in the levels of CB_1_ receptors between the autoradiography and Western blot study is probably related to the internalization and trafficking of these receptors (Coutts et al. 2001; Leterrier et al. 2004). These molecular processes could explain the lack of changes in the expression of CB_1_ receptors analyzed in homogenates of the whole structure (membrane and endogenous receptors), while changes were detected in the CB_1_ receptor density after binding the tritiated agonist to the membrane receptors with constitutive activity during the autoradiography. Furthermore, the bulbectomy-induced increase in CB_1_-receptor signaling in the prefrontal cortex was reversed by chronic fluoxetine administration (Rodriguez-Gaztelumendi et al. 2009).

Our present findings also demonstrated a reduction in AEA levels in the hippocampus in the OBX rats accompanied by increased FAAH expression. AEA is metabolized primarily by FAAH, and the reduced AEA levels may impair neurogenesis in the hippocampus in the OBX rats. In fact, it was reported that hippocampal neurogenesis was reduced in depressed patients (Sheline et al. 1996) and in animal models of depression (Toth et al. 2008) probably due to AEA-mediated excitotoxic damage (Marsicano et al. 2003). In fact, our previous study showed that hippocampal glutamate levels are elevated in OBX rats (Jastrzebska et al. 2015). Antidepressant-like effects have been demonstrated after activation of the eCB system in the hippocampus following either FAAH inhibition (Bambico et al. 2010) or direct CB_1_ receptor stimulation (McLaughlin et al. 2007). Altogether, the dampened hippocampal eCB signaling seen in our study may provoke the opposite, i.e., depression-like, effects. Our current findings are strongly supported by studies using several animal models of depression based on stress (Hill et al. 2008a; Dubreucq et al. 2012). In the present study, both down-regulation of CB_1_ and CB_2_ receptors and a decrease in the AEA level were present in the hippocampus after bulbectomy. Administration of AEA for 5 days resulted in a significant increase in CB_1_ receptor density in the hippocampus (Romero et al. 1995), so moderate activation of CB_1_ receptor activity can result in its up-regulation in the hippocampus, while by analogy, a bulbectomy-induced decrease in AEA level provokes the decrease in CB_1_ receptor density due to withdrawal of a trophic factor (ligand) for CB_1_ receptor expression (Hill et al. 2005). Additionally, synthesis of eCBs is modulated by changes in other neurotransmitter systems (Smaga et al. 2014a, b), so it is difficult to speculate on the exact mechanism of action of these physiological changes. However, in opposition to Vinod et al. (2012), we did not detect changes in the AEA level in the hippocampus in the WKY rats. The reasons for these differences between the present and Vinod et al. (2012) results are probably related to the different conditions used during eCB detection. Despite the lack of changes in the levels of endogenous ligands in this study, similar increases in the CB_1_ receptor expression and an increase in the density of these receptors in the hippocampal dentate gyrus and CA1 and CA3 fields were noted in the WKY rats (present study; Vinod et al. 2012).

Reduction in the AEA levels was also seen in the striatum in both the OBX and WKY rats. The latter change seems to be associated with a weaker hedonic response (anhedonia), a symptom characteristic of a depressive state (Hill et al. 2008a). Several studies have shown that attenuation of the striatal eCB signaling provoked depressive-like behavior in stress-related models (i.e., CUS and chronic mild stress (CMS)) (Hill et al. 2008a; Reich et al. 2013a, b; Segev et al. 2013) and in OBX rats (Eisenstein et al. 2010), while several antidepressant drugs increased the striatal AEA contents (Smaga et al. 2014a). The weakening of the eCB signaling in the OBX rats was supported also by a decrease in the density of CB_1_ receptors in the laterodorsal striatum and by the lowered CB_1_ receptor expression in the dorsal striatum. The data in the literature on rats with depressed phenotype (after CUS procedure) confirm our observations (Hill et al. 2008a). Interestingly, in contrast to bulbectomy-induced changes, an increase in the CB_1_ receptor density in the laterodorsal striatum together with up-regulation of CB_2_ receptors were observed in this structure in the WKY rats.

The reduced AEA levels in this structure may be either caused by the increased expression of FAAH seen in the WKY rats (but not in the OBX rats) or may be related to the decreased eCB release regulated by synaptic activity. In fact, the level of neuronal firing modulates postsynaptic eCB release from postsynaptic neurons and long-term depression (LTD) at both glutamatergic and GABAergic synapses in the striatum (Adermark et al. 2009). Endogenous dopamine is also involved in the generation of eCB-LTD by synaptic activity via the striatal D_2_ receptors (Kreitzer and Malenka 2005), and in addition, altered dopaminergic neurotransmission has been shown to provoke anhedonia (Holmes 1999). Bulbectomy induces an increase in dopamine release and D_1_ and D_2_ receptor gene expression in the striatum (Holmes 1999; Masini et al. 2004), while alterations of this neurotransmitter evokes a decrease in the AEA release via D_1_ receptors (Patel et al. 2003), which was also observed in the OBX rats in the present study. Moreover, the increased locomotor activity seen in this paper was probably related to up-regulated dopamine release in the striatum, which was also confirmed in another study (Masini et al. 2004).

The opposing regulation of AEA and 2-AG tissue contents was observed in the rat nucleus accumbens, where the AEA levels were elevated, while the 2-AG concentration decreased after bulbectomy. Interestingly, expression of the AEA synthesizing enzyme and the 2-AG degrading enzyme was increased, which can explain the changes in the eCB levels in the OBX rats. On the other hand, in the WKY rats, although there was a decrease in the expression of the enzymes synthesizing and degrading AEA, homeostasis of this mediator was not compromised, and the AEA levels did not change in the nucleus accumbens. Lack of changes in the levels of neurotransmitters, despite disturbances in metabolizing enzymes, may be associated with a local increase in the density and expression of CB_1_ receptors in the core and shell of the nucleus accumbens in the WKY rats.

Changes in the eCB levels in the nucleus accumbens in the OBX rats seem to be closely related to alterations in other brain neurotransmitters. eCBs are engaged in the LTD of glutamatergic signaling in the nucleus accumbens. The glutamatergic synapses, where CB_1_ receptors are located, modulate the firing of GABAergic neurons in the nucleus accumbens, which in turn decreases the dopaminergic neurons of the ventral tegmental area (VTA). eCBs may disinhibit dopamine cells of the VTA by reducing the excitatory transmission in the nucleus accumbens, increasing the dopamine firing rate, and provoking dopamine release in the nucleus accumbens (Robbe et al. 2001). In the OBX rats, it was shown that glutamatergic and GABAergic neurotransmission was dysregulated, which also led to the changes in the dopaminergic system (Song and Leonard 2005; Jastrzebska et al. 2015). Changes in the OBX rats were accompanied by reduced expression of CB_1_ receptors in the nucleus accumbens, but this change does not seem to be related to the levels of neurotransmitters. Additionally, several papers showed that AEA administration, an FAAH inhibitor or CB_1_ agonists evoked augmentation of dopamine concentration in the nucleus accumbens shell in rats (Hungund et al. 2003; Lecca et al. 2006; Solinas et al. 2006; Cadoni et al. 2008) as well as self-administered 2-AG stimulated dopamine release in the nucleus accumbens shell in rats (De Luca et al. 2014).

Finally, changes in the eCB system were also detected in the cerebellum only in the WKY rats, while no changes in the eCB levels and in the expression of enzymes involved in their metabolism were observed. In addition, an increase in the expression of CB_1_ and CB_2_ receptors was noted in the cerebellum in the WKY rats. In naïve rats, the CB_1_ receptors are strongly expressed on basket cells located in the cerebellar cortex, where they form synapses on the cell bodies of Purkinje cells and make inhibitory synapses with Purkinje cells (Yoshida et al. 2002). Stimulation of the CB_1_ receptor suppresses spontaneous inhibitory postsynaptic currents (IPSCs) and is involved in the depolarization-induced suppression of inhibition (DSI) by influencing the presynaptic inhibitory terminals (Yoshida et al. 2002). The strengthened eCB signaling by up-regulation of CB receptors, observed in our study, can aggravate inhibitory signals from GABA terminals in the cerebellum and induce a depression-like phenotype in the WKY rats.

## Conclusion

In summary, the common change observed in both the genetic (WKY) and bulbectomy-induced models of depression entails weakness of eCB signaling (AEA) in the dorsal striatum, what can induce a characteristic symptom of depression, anhedonia. In other brain structures, neurochemical changes were different, and they seemed to be associated with the particular brain structures and the factors inducing depression. For the first time, our study showed changes in the CB_2_ receptor expression in several brain structures in animal models of depression and the importance of alterations in eCB metabolizing enzymes as a potential factor inducing changes in eCB levels. The changes in the eCB system observed in several rat brain structures in different animal models of depression suggest a complex role of this system in the pathogenesis of depression.

## Abbreviations

2-AG: 2-arachidonoylglycerol
5-HT: serotonin
AEA: anandamide
CB: cannabinoid
DAGLα: diacylglycerol lipase α
eCB: endocannabinoid
FAAH: fatty acid amide hydrolase
LC-MS/MS: liquid chromatography tandem mass spectrometry
MAGL: monoacylglycerol lipase
NAPE-PLD: N-acyl phosphatidylethanolamine phospholipase D
OBX rats: bulbectomized rats
WKY rats: Wistar Kyoto rats
